# A SnO_2_/CeO_2_ Nano-Composite Catalyst for Alizarin Dye Removal from Aqueous Solutions

**DOI:** 10.3390/nano10020254

**Published:** 2020-02-01

**Authors:** Saad S. M. Hassan, Ayman H. Kamel, Amr A. Hassan, Abd El-Galil E. Amr, Heba Abd El-Naby, Elsayed A. Elsayed

**Affiliations:** 1 Chemistry Department, Faculty of Science, Ain Shams University, Abbasia 11566, Cairo, Egypt; amr_hassan@sci.asu.edu.eg (A.A.H.); hoba_science@hotmail.com (H.A.E.-N.); 2 Department of Chemistry, Virginia Commonwealth University, Richmond, VA 23284, USA; 3 Pharmaceutical Chemistry Department, Drug Exploration & Development Chair (DEDC), College of Pharmacy, King Saud University, Riyadh 11451, Saudi Arabia; 4 Applied Organic Chemistry Department, National Research Center, Dokki 12622, Giza, Egypt; 5 Bioproducts Research Department, Zoology Department, Faculty of Science, King Saud University, Riyadh 11451, Saudi Arabia; eaelsayed@ksu.edu.sa; 6 Chemistry of Natural and Microbial Products Department, National Research Centre, Dokki 12622, Cairo, Egypt

**Keywords:** tin oxide/cerium oxide, nano-composite, adsorption of dyes, alizarin dyes removal

## Abstract

A new SnO_2_/CeO_2_ nano-composite catalyst was synthesized, characterized and used for the removal of alizarin dyes from aqueous solutions. The composite material was prepared using a precipitation method. X-ray powder diffractometry (XRD), high resolution transmission electron microscopy (HR-TEM), Brunauer–Emmett–Teller methodology (BET) and Fourier Transform Infrared Spectrometry (ATR-FTIR) were utilized for the characterization of the prepared composite. The prepared nano-composite revealed high affinity for the adsorption and decomposition of alizarin dyes. The adsorption capacity under different experimental conditions (adsorbate concentration, contact time, adsorbent dose and pH) was examined. Under optimized experimental conditions, the removal of alizarin yellow, alizarin red and alizarin-3-methylimino-diacetic acid dyes from aqueous solutions was about 96.4%,87.8% and 97.3%, respectively. The adsorption isotherms agreed with the models of Langmuir, Freundlich and Temkin isotherms.

## 1. Introduction

The lack of water resources requires humanity to save each drop of it, but unfortunately there are plenty of pollutants that may affect the quality of the water resources. The contamination of water resources with dyes is an important source of pollution. Many industries use dyes during processing of their products such as textiles, dyestuff, distilleries, tanneries, paper, rubber, plastics, leather, cosmetics, food and pharmaceuticals for the coloration of their products. Hence, effluents from these industries commonly contain dye residue. Millions of tons of dye effluent are dumped into water bodies and cause environmental problems [1]. 

Alizarin dye (1,2-dihydroxy-9,10 anthraquinone sulfonic acid sodium salt) is a pollutant, released chiefly by textile industries. Suspected carcinogenic effects of this dye make it important to remove it from water [2,3,4]. Membrane separation, ion-exchange methods [5,6], photocatalysis [7,8,9,10], the electro-Fenton process [11] and adsorption methods [12,13,14,15,16,17,18,19,20,21] are commonly used techniques for the removal of this group of dyes.

Nano-technology offers an opportunity to develop adsorbents with large surface area [22], high adsorption capacity, high removal rate and large number of active surface sites [23]. In recent years, metal oxide nano-composites have attracted considerable attention for energy and environmental applications because of their ease of fabrication, low processing cost and, particularly, that the presence of nano-grains with various sizes and various kinds of nano-composite materials have been investigated for efficient removal of different organic pollutants. For example, either SnO_2_or CeO_2_nano-particles (NPs) coupled with some metal oxides such as TiO_2_ [24], ZnO [25], CeO_2_ [26], MnO_2_ [27] and Fe_3_O_4_ [28] are used as for the removal of dyes.

In the present work, SnO_2_ doped with CeO_2_nano-particles was prepared using a simple and cost effective co-precipitation method at room temperature and utilized for the removal of some alizarin dyes. The composite was characterized by different spectrochemical techniques. Physical parameters affecting maximum dye removal were examined.

## 2. Materials and Methods 

### 2.1. Materials

All chemicals used were of analytical reagent grade. Alizarin red S was purchased from Riedel-Dehaën (Delhi, India), and alizarin-3-methylimino-diacetic acid was from Fluka (Ronkonoma, NY, USA). Ceric ammonium nitrate (NH_4_)_2_Ce(NO_3_)_6_ was from SDFCL (Delhi, India). Ammonium hydroxide and tin (IV) chloride pentahydrate SnCl_4_.5H_2_O were from ADWIC (Cairo, Egypt). Polyethylene glycol 6000 was from Merck (St. Lois, MO, USA). All chemicals were used as received without any further purification.

### 2.2. Preparation of SnO_2_/CeO_2_ Nano-Composite Catalyst

Three and one-half grams of SnCl_4_·5H_2_O and 0.274 g of (NH_4_)_2_Ce (NO_3_)_6_ were dissolved in 100 mL distilled water followed by addition of 1.0 g polyethylene glycol (PEG) as a stabilizing agent. The mixture was kept under magnetic stirring for one hour. To the above mixture, ammonia solution (35.04 v/v%) was added drop-wise with vigorous stirring until the solution reached pH 9. The mixture was kept under magnetic stirring for another two hours and then left overnight. The precipitate was filtered off and washed with distilled water until the washings were free from chloride ion. The obtained yellowish precipitate was then dried at 70 °C for two hours and calcined at 900 °C in air for three hours.

### 2.3. Characterization of Nano-Composite Catalyst

The prepared composite was characterized using high-resolution transmission electron microscopy (HR-TEM) using Jeol 2100 (Osaka, Japan), X-ray powder diffractometry (XRD) using X’Pert PRO, PANalytical (Amsterdam, The Netherlands) with CuKα radiation (*λ* = 0.154060 nm) in the angular region of 2*θ* = 4–80° operated at 40 KV and 40 mA. The spectra were recorded at a scanning speed of 8° min^−1^. A Nicolet™ iS50 (ATR-FTIR) spectrometer was used in a spectral range of 4000–500 cm^−1^. The Brunauer–Emmett–Teller (BET) surface area measurements were carried out by nitrogen adsorption–desorption at 77 K using NOVA 3200s (Florida, CA, USA), at the relative pressure (*P/Po*) of 0.95104.

### 2.4. Dye Uptake Study

Fifteen milligrams of the adsorbent (SnO_2_/CeO_2_ nano-composite) was added to 30 mL aliquots of alizarin dye solutions (25, 30, 40, 50, 60, 70, 80 and 90 µg/mL). The solutions were stirred for 30 min at room temperature and filtered. Dye concentrations, before and after the treatment, were measured spectrophotometrically at *λ_max_* (422 or 515 nm) in an acidic and alkaline medium, respectively. The removal percentage of the dye was calculated using the following equation:

Removal % = [(*C*_0_ − *C_t_*)/*C*_0_] × 100 (1)

where *C_0_* and *C_t_* are the dye concentration in mg/L at initial and after time *t*, respectively. 

### 2.5. Adsorption Studies

Aliquots (30 mL) of varying concentrations (25–90 mg/L) of the dye solution were treated with different adsorbent doses (0.01–0.2 gm), under different pH values (2–9) for varying contact times (5–180 min). The dye solutions were stirred and filtered, and the dye concentration was spectrophotometrically measured at either *λ_max_* 422 or 515 nm in the pH range 2–5 and pH range 6–9, respectively. 

## 3. Results and Discussions

### 3.1. Characterization of the Nano-Composite Catalyst

#### 3.1.1. X-ray Diffraction Pattern of SnO_2_/CeO_2_ Nano-Composite

**Figure 1:**
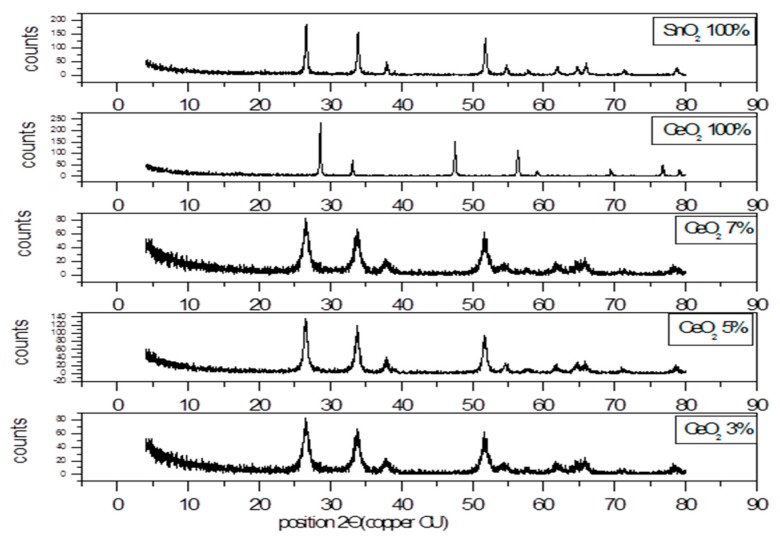
XRD pattern of SnO_2_/CeO_2_ nano-particles (NPs).

Tin oxide/cerium oxide (SnO_2_/CeO_2_) nano-composite materials consisting of 93–97% SnO_2_ and 7–3% CeO_2_ were prepared and characterized. The XRD pattern (Figure 1) of the pure SnO_2_ and CeO_2_ showed peaks matched with the diffraction data of the tetragonal structure of tin oxide (JCPDS 04-008-8131) and of the cubic structure of CeO_2_ (JCPDS 00-033–0334). On the other hand, the composites showed main diffraction patterns at 26.4°, 34.3° and 52.4° corresponding to (110), (101) and (211) of SnO_2_. No characteristic peaks of the CeO_2_ composite appeared in the XRD spectrum due to the small amount of this oxide in the composite. The mean crystallite size (*D*) of the nano-particles was calculated using the Debye–Scherrer formula [29] (Equation (2)).

*D* = *Kλ*/*β*cos*θ* (2)

where *K* and *λ* are the Scherrer constant and the X-ray wavelength of radiation used (*Kα* − Cu = 0.154060 nm), respectively. The constants *β* and *θ* are the full width at half maximum (FWHM) of diffraction peak and the Bragg diffraction angle, respectively. Since the position of the main peak is (2*θ* = 26.42) and the width of the peak is 0.3149 nm, the crystallite size (*D*) is about 27 nm SnO_2_ doped with 5% CeO_2_. The mean crystallite size (*D*) of SnO_2_ doped with 3% and 7% CeO_2_ nano-particles was estimated to be 18.06 nm.

#### 3.1.2. High-Resolution Transmission Electron Microscopy (HR-TEM) of SnO_2_/CeO_2_ Nano-Composite

**Figure 2:**
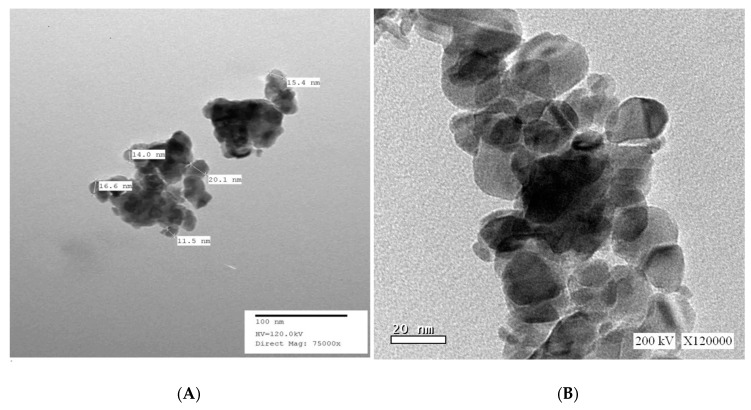
TEM images of SnO_2_/CeO_2_ nano-composite (**A**): magnification of ×75,000 and (**B**): magnification of ×120,000.

The morphology and average particle size of the prepared SnO_2_/CeO_2_ (95%:5%) nano-composites were examined by high-resolution transmission electron microscopy (HR-TEM). As shown in Figure 2, it indicates that SnO_2_/CeO_2_ NPs have spherical morphologies with particle sizes varying from 11.5 to 30 nm, which is in agreement with the crystallite size obtained from XRD data.

#### 3.1.3. Fourier Transform Infrared Spectroscopy of SnO_2_/CeO_2_ Nano-Composite

**Figure 3:**
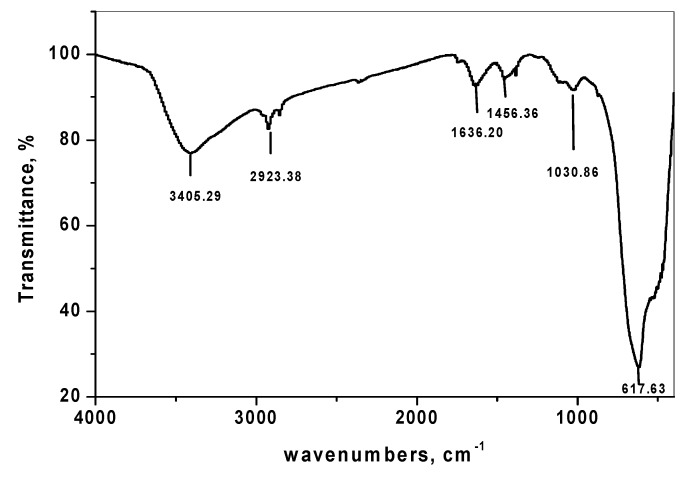
FTIR spectrum of SnO_2_/CeO_2_ NPs.

The FTIR spectrum of SnO_2_/CeO_2_ nano-particles is shown in Figure 3. The characteristic peak at 3405.29 cm^−1^ was due to the stretching vibration of the O–H bond of the physically adsorbed water molecule on the SnO_2_/CeO_2_ surface [24]. The peak appearing presented at 1636.20 cm^−1^ was due to the bending vibration of the adsorbed water molecule, and the displayed peak at 617.63 cm^−1^ was due to the stretching modes of the M–O bond.

#### 3.1.4. Porous Structure

**Figure 4:**
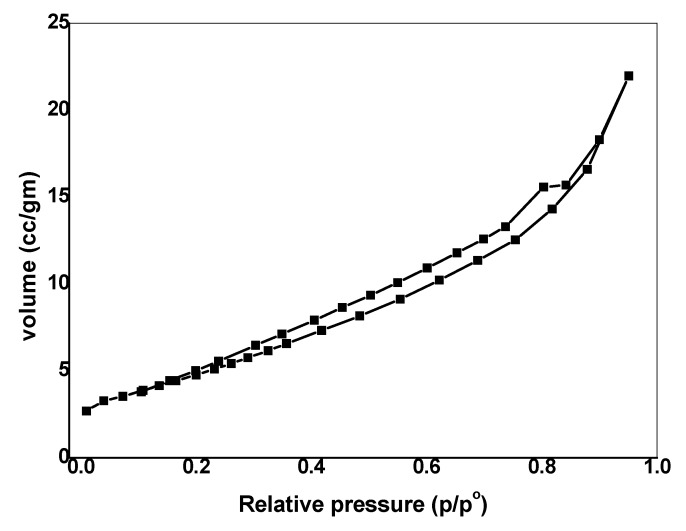
N_2_ adsorption–desorption isotherms of coupled SnO_2_/CeO_2_ NPs.

The Brunauer–Emmett–Teller (BET) method was used for investigating the pore structure of the prepared nano-composite material. The nitrogen adsorption–desorption experiment of SnO_2_/CeO_2_ nano-composite gives the isotherms depicted in Figure 4, which is of the type IV [30] with mesoporous structure. The BET surface area and pore volume of the nano-composite are shown in Table 1.

#### 3.1.5. Electrocatalytic Behavior of SnO_2_/CeO_2_ Nano-Composite

**Figure 5:**
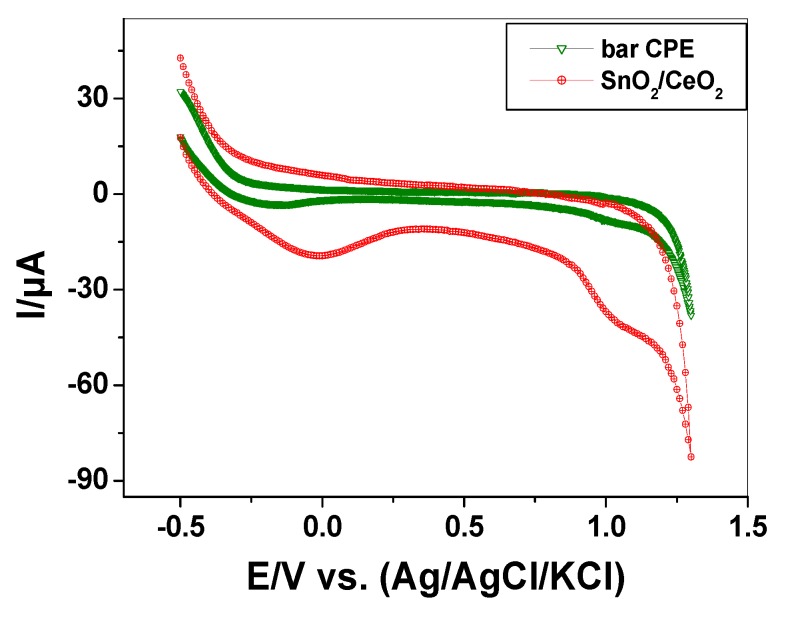
Cyclic voltammograms of carbon past electrode (CPE) and SnO_2_/CeO_2_/CP electrode at a scanning rate of 300 mV/s.

Voltammetric measurements were performed to prove the electrocatalytic effect of the nano-composite SnO_2_/CeO_2_ using a potentiostat (Model 273A Princeton Applied Research (PAR), Princeton, Oak Ridge, TN, USA). Three electrode cell was used for voltammetric measurements containing an Ag/AgCl/KCl(s) reference electrode (BAS Model MF-2063, BASi, West Lafayette, OH, USA), and a platinum wire (BAS Model MW-1032) was used as a counter electrode. The working electrode was a Teflon rod with an end cavity (3 mm diameter and 5 mm deep) bored at one end for paste filling (BASi-MF-2010, West Lafayette, OH, USA) and connected with a copper wire through the center of the rod [31]. The carbon paste (CP) was prepared by thoroughly hand mixing 0.05 g SnO_2_/CeO_2_ nano-composite and 0.95 g of graphite powder with 360 µL of nujol oil in an agate mortar with pestle to give a homogenous 5% (w/w) SnO_2_/CeO_2_/CP. The cyclic voltammograms of SnO_2_/CeO_2_/CP electrode is shown in Figure 5 in a scan range of −0.5 to 1.5 V at a scanning rate of 300 mV/s. The peaks obtained correspond to the surface reactions, where Sn^4+^ and Ce^4+^ were reduced to Sn^2+^ and Ce^3+^, respectively. Table 2 shows the redox potentials (E) of cerium, tin and alizarin. As shown in Figure 5, the reduction peaks that appeared at *E_sn_ =* −0.03 V and *E_ce_* = 1.0 V reflect the electrocatalytic effect of the catalyst and its capability to oxidize alizarin dye in aqueous solutions (*E_ALZ_* = −0.59).

### 3.2. Use of SnO_2_/CeO_2_ Nano-Composite for Alizarin Dye Removal

**Figure 6:**
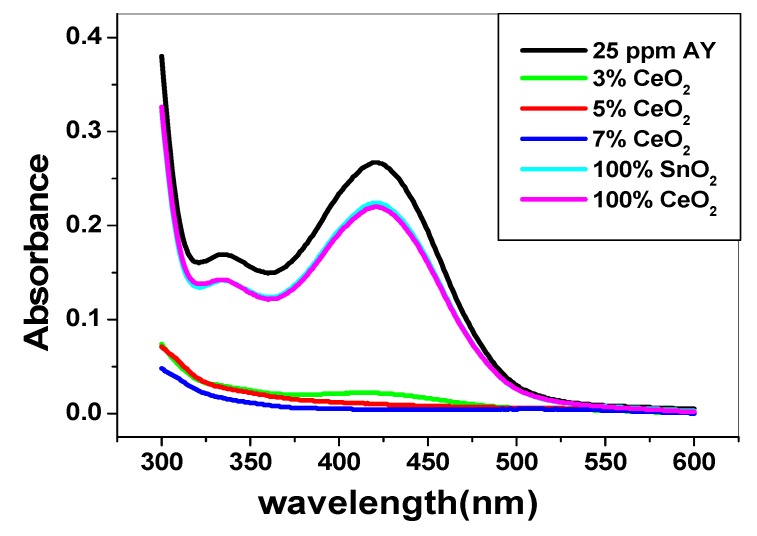
UV spectra of alizarin dye subjected to removal with different CeO_2_ % at *λ_max_* = 422 nm.

The effect of CeO_2_ amount doped with SnO_2_ on the removal efficiency was tested. Three different nano-composites with a percentage amount of CeO_2_of 3%, 5% and 7% (w/w) were chosen for alizarin removal. It was found that as the amount of CeO_2_ doped increased in the composite, the removal percentage increased to be 91.3%, 96.4% and 98.3% for 3%, 5% and 7% CeO_2_, doped with SnO_2_, respectively. This implies that CeO_2_ enhanced the adsorption and electrocatalytic powers of the synthesized nano-composite. As seen in Figure 6, pure SnO_2_ and CeO_2_ had no catalytic degradation on alizarin dyes. This reflects the enhanced catalytic power of the synthesized nano-composites. 

#### 3.2.1. Effect of pH and Catalytic Activity of SnO_2_/CeO_2_ Nano-Composite

**Figure 7:**
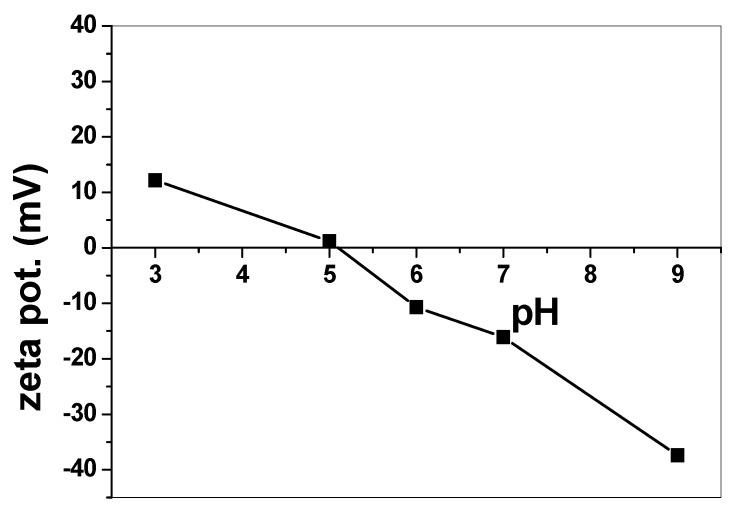
Zeta potential of SnO_2_/CeO_2_ nano-catalyst at different pHs.

Zeta potential was measured to interpret the behavior of SnO_2_/CeO_2_ nano-composite at different pH values. Figure 7 shows that in an acidic medium (below pH 5), the catalyst surface became positively charged. This could enhance the adsorption via electrostatic attraction between alizarin dye and the catalyst. The zero point charge of the catalyst at pH 5–6 led to no or very slight alizarin removal. In alkaline media, the catalyst surface became negatively charged, and a gradual decrease in alizarin removal was noticed. 

**Figure 8:**
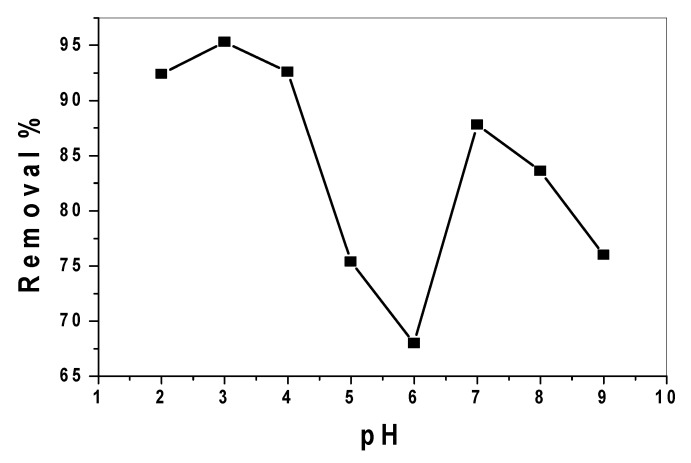
Effect of pH on alizarin removal (V = 30 mL, AY conc. = 25 µg/mL, contact time = 30 min and catalyst amount = 0.15 g).

To optimize the pH under which maximum removal of alizarin took place, a 30 mL aliquot of alizarin dye solution containing 25mg L^−1^ was treated with 0.15 g of the adsorbent at pH values ranging from 2 to 9 and was stirred for 30 min. A maximum removal of alizarin with the value 95.3% was detected at pH 3.0 and then decreased to 68.0% at pH 6.0. A further increase in the removal efficiency 87.8% was detected at pH 6.5–7.5, and then it began to decline after further increase in the pH values (Figure 8). It has been reported that tin (IV) oxide is predominately a Lewis acid, with weak Bronsted acidity evolving in the presence of water vapor. Strong Bronsted-acid sites can be produced by protonation in acidic media. Additionally, more adsorption at acidic pH indicates that the lower pH results in an increase in H^+^ ions on the adsorbent surface that result in significantly strong electrostatic attraction between alizarin molecules and the adsorbent surface.

In addition, when the catalyst was irradiated by the solar light, electrons from the conduction band and holes from the valance band were formed [25], generating the primary oxidant, hydroxyl radicals (OH^•^), due to the reaction between hydroxide ions and positive holes. The hydroxyl radicals are considered the major oxidation species in an alkaline pH medium. At pH levels greater than 7.0, there was a decrease in alizarin removal due to the electrostatic repulsion of the negatively charged surface of the nano-composite and hydroxide ions, which in turn prevented the formation of OH^•^ radicals (primary oxidant). So, it can be concluded that the high removal percentage implies the occurrence of physical and chemical adsorption in acidic solutions and chemical degradation in alkaline solutions.

#### 3.2.2. Effect of Contact Time

**Figure 9:**
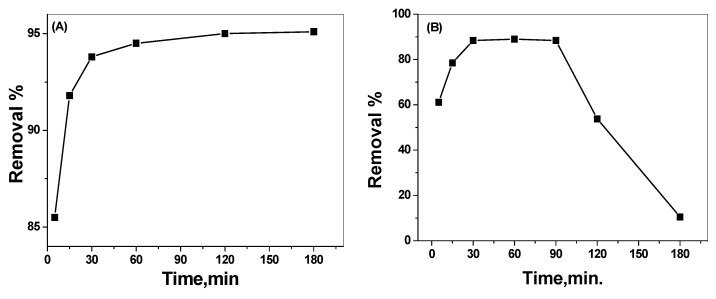
Effect of contact time on alizarin removal at (**A**) pH 3.0, (**B**) pH 7.0 (V = 30 mL, AY conc. = 25 µg/mL and catalyst amount = 0.15 g).

The optimal time required for maximum dye removal was tested by fixing all other parameters (30 mL of 25 mg/L alizarin dye (AY), pH 3, 7 and 0.15 g of the nano-composite) and varying the contact time from 5 to 180 min. At pH 3, the dye removal increased from 85.5% after a 5 min contact time to 94% after 30 min (Figure 9A). Further increase of the contact time over 30 min had no noticeable effect for the removal of more dye concentration. At pH 7, the removal of the dye increased from 60% to 91.1% for the above mentioned contact time (Figure 9B). Further increase of the contact time over 30 min led to a rapid decrease in the removal efficiency. This can be attributed to the electrostatic repulsion between the negatively charged surface present on the adsorpant at this pH and alizarin molecules. A contact time of 30 min was chosen for all subsequent measurements. 

#### 3.2.3. Effect of the Catalyst Dose 

**Figure 10:**
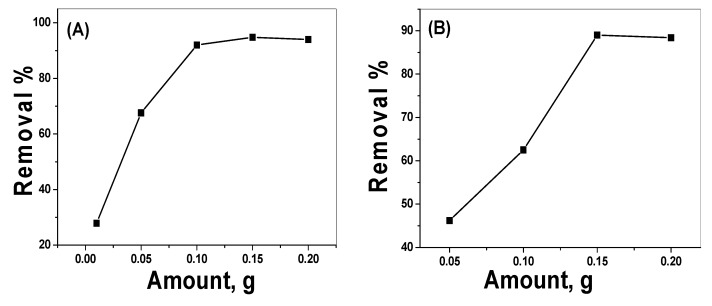
Effect of adsorbent amount on alizarin removal at (**A**) pH 3.0, (**B**) pH 7.0 (V = 30 mL, AY conc. = 25 µg/mL and contact time = 30 min).

Different quantities of NP material (0.01 to 0.2 g) were mixed with 30 mL (25 mg/L) alizarin dye solution at pH 3.0 and stirred for 30 min. Figure 10A shows that the removal of the dye increased from 28% with 0.01 g adsorbent to 94.8% for 0.15 g of adsorbent. Under similar conditions, but at pH 7, 46.2% and 88.8% removal efficiency were obtained (Figure 10B). An adsorbent quantity of 0.15 g was used for further study.

#### 3.2.4. Effect of Alizarin Dye Concentration

**Figure 11:**
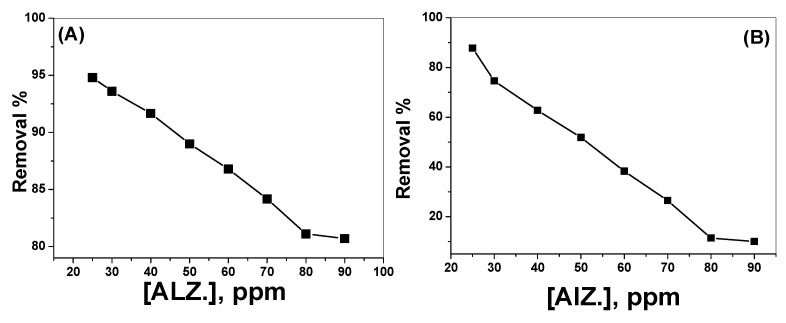
Effect of alizarin dye concentration on the removal of the dye at (**A**) pH 3.0, (**B**) pH 7.0 (V = 30 mL, contact time = 30 min and catalyst amount = 0.15g).

The effect of dye concentration in the range from 25 to 90 mg/L at pH 3 and 7 was investigated under the optimized experimental parameters. Thirty milliliter aliquots of alizarin yellow dye solution at both pH 3.0 and 7.0 were allowed to interact with 0.15 gm SnO_2_/CeO_2_ nano-composite for 30 min. It was noticed that the removal percentage decreased as the concentration of the dye increased from 25 to 90 mg/L. At pH 3, 94.8% and 81% alizarin removal was obtained with 25 mg/L and 80 mg/L alizarin concentration, respectively (Figure 11A). At pH 7, the removal efficiency decreased from 87.8% to 11.4% when the concentration of the dye increased from 25 mg/L to 80 mg/L, respectively (Figure 11B).

### 3.3. Removal of Alizarin-3-Methylimino-Diacetic Acid (AMA)

Under the previously optimal experimental conditions, 95.0% of alizarin-3-methylimino-diacetic acid dye was removed, confirming the efficiency of SnO_2_/CeO_2_ nano-composite for the removal of different alizarin group dyes.

### 3.4. Modeling of Adsorption

Langmuir (Equation (3)), Freundlich (Equation (4)) and Temkin (Equation (5)) models were applied to calculate the sorption of alizarin dye.

*1/Q_t_ = 1/X_m_bC_t_ + 1/X_m_* (3)

*LogQ_t_ = (1/n) log C_t_ + log k_F_* (4)

**Figure 12:**
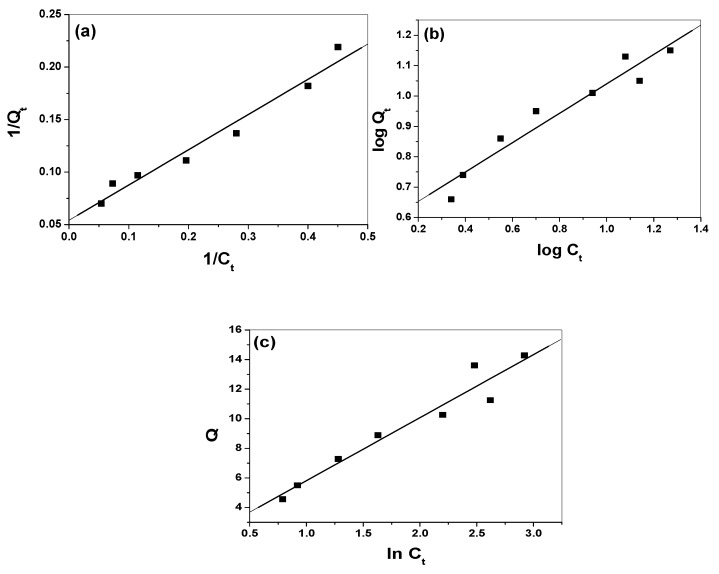
(**a**) Langmuir, (**b**) Freundlich and (**c**) Temkin isotherms for alizarin removal.

*Q_t_ = (RT/B_T_) ln C_t_ + (RT/B_T_) lnK_T_* (5)

where *Q_t_* is the adsorption capacity at equilibrium (mg/g); *C_t_* is the equilibrium concentration of the AY solution (mg/L); *t* (min) is the contact time; *X_m_* (mg/g) is the maximum monolayer adsorption capacity and *b* (L/mg) is the adsorption equilibrium constant. A linear relationship was obtained by plotting *1/Qtversus1/C_t_* (Figure 12a). The slope (*X_m_*) and the intercept (*b*) values were 18.52 and 0.16, respectively, suggesting monolayer adsorption with correlation coefficient (*R^2^* = 0.983).

From the Freundlich model, the relative adsorption capacities (*n*) and sorption intensities *(K_f_*) (mg/gm) were calculated from the slope and intercept of plotting *logQ_t_* vs. *log C_t_* (Figure 12b). The values were *n* = 2.08, indicating a favorable sorption process as *n* > 1 [34], and *K_f_* = 3.55 mg/g with a correlation coefficient (*R^2^* = 0.966). Temkin constants, *B_T_* (k J/mol) and *K_T_* (L/mg), which are constants of heat of sorption and the equilibrium binding constant corresponding to maximum binding energy, were estimated from the slope and intercept of *Q_t_* vs. ln *C_t_* (Figure 12c). Values of 0.533 and 1.44 were obtained, respectively. This refers to the heat of sorption and confirms the physical adsorption process with a correlation coefficient (*R^2^* = 0.970). All of these values confirmed a reasonable adsorption capacity and suggested the occurrence of both physical (multilayer) and chemical (monolayer) adsorption [35] between the prepared nano-composite and alizarin dye that confirms the results obtained from the pH effect.

### 3.5. Regeneration of SnO_2_/CeO_2_ Nano-Composite

The adsorbent was regenerated after each adsorption cycle of alizarin by heating at 600 °C for 60 min. After five cycles of regeneration, the efficiency of the nano-composite for the removal of alizarin dye became 77.8%.

### 3.6. Comparison with Other Sorbents for Removal of Alizarin

Table 3 represents a comparison of the performance of the present suggested sorbent with some of those previously described nano-materials for the removal of alizarin veiled. It can be seen from Table 3 that high maximum adsorption capacity [7], reasonable contact time [7,13,14,18,19,36,37,38,39,40,41], high removal percentage [7,8,10,14,15,18,36,38,39,41] and low amount of adsorbent dosage [7,8,10,15,38] were offered by the present suggested nano-composite material in this work.

## 4. Conclusions

A facile method was presented for the removal of alizarin dyes from aqueous industrial effluents. The method was based on the use of SnO_2_/CeO_2_ nano-composite catalyst. The catalyst was synthesized by the so-called “co-precipitation method” and characterized by X-ray powder diffractometry (XRD), high-resolution transmission electron microscopy (HR-TEM), Brunauer–Emmett–Teller methodology (BET) and Fourier transform infrared spectrometry (ATR-FTIR). Polycrystalline and spherical structure were confirmed with a mean average grain size of 27 nm. The prepared nano-composite revealed a high affinity for the adsorption and decomposition of alizarin dyes. Alizarin-3-methylimino-diacetic acid, alizarin yellow and alizarin red S dyes showed removal efficiencies of 95.0%, 95.3% and 87.8%, respectively. The optimal conditions for the adsorption capacity were pH 3.0, 25 mg/L dye, 30 min contact time and 0.15 gm catalyst. The adsorption isotherms agreed with Langmuir, Freundlich and Temkin isotherms. A comparison of the performance of the present suggested sorbent with some of those previously described nano-materials for the removal of alizarin is shownin Table 3. The presented method offered high maximum adsorption capacity, reasonable contact time, high removal percentage and low amount of adsorbent dosage compared to those presented by the previously reported methods.

## Figures and Tables

**Table 1:** General surface characteristics of SnO_2_/CeO_2_ nano-composite obtained byN_2_ adsorption at 77 K.

Sample	Surface Area (m^2^ g^−1^)	Average Pore Volume, (cm^3^ g^−1^)	Average Pore Diameter (nm)
SnO_2_/CeO_2_ NPs	18.970	3.399 × 10^−2^	7.164

**Table 2:** Redox potentials (*E*) of cerium, tin and alizarin.

System	Standard Redox Potential, *E°* (V)	Ref.
Ce^4+^(aq) + e^−^ 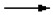 Ce^3+^(aq)	+1.61	[32]
Sn^4+^(aq) + 2 e^−^ 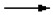 Sn^2+^(aq)	+0.15	
Sn^2+^(aq) + 2 e^−^ 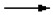 Sn(s)	−0.14	
Alizarin 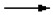 Alizarin oxidation product	−0.59	[33]

**Table 3:** Comparison of various nano-adsorbents for alizarin dyes removal.

Adsorbent Type	Maximum Adsorption Capacity, mg/g	Contact Time	Optimum pH	Best Fit Isotherm	Adsorbent Dosage, gm	Removal, %	Ref.
ZnO/TiO_2_	12.5	120 min	8	Langmuir	5.0	84.4–92.9	[7]
Zinc doped WO_3_ catalyst	NR	10 min	NR	NR	0.4	80	[8]
α-Fe_2_O_3_/NiS	NR	NR	5	NR	1.0	88.3	[10]
Activated carbon/γ-Fe_2_O_3_ nano-composite	108.6	60 min	2	Langmuir	0.01	99.4	[13]
Poly METAC/Fe_3_O_4_ magnetic nanoparticles	NR	2 days	NR	NR	NR	80–96	[14]
Nanocrystalline Cu_0.5_Zn_0.5_Ce_3_O_5_	NR	5 min	NR	Freundlich	0.2	83%	[15]
PPy-coated Fe_3_O_4_ nanoparticles	116.3	60 min	4–5.4	Langmuir	0.1–0.12	78.7	[18]
Chitosan-coated Fe_3_O_4_ nanoparticles	40.12	50 min	3	Langmuir	0.1	NR	[19]
2,4-dinitrophenyl hydrazine/Nano 𝛾-Alumina	47.8	60 min	4	Langmuir	0.05	95.6	[36]
MWCNTs/PANI	884.8	50 min	8.5	Langmuir	0.02	N.R	[37]
Fe_3_O_4_ nanoparticles	45.8	5 min	5	Langmuir	0.02	99	[42]
CuFe_2_O_4_@graphene nanocomposite	145	40 min	3	Langmuir	0.5	95	[38]
Chitosan/ZnO nanorod composite	36.4	27 h	2	Freundlich	0.1	85	[39]
Fe_3_O_4_@MCM@Cu–Fe–LDH	121.9	10 min	9	Langmuir	0.03	N.R	[43]
Biosorbent from Mikaniamicrantha	46.5	N.R	2	Freundlich	0.1	N.R	[40]
Green carbon composite-derived polymer resin and waste cotton fibers	104	1day	3	Freundlich	NR	N.R	[44]
NiFe_2_O_4_/Polyaniline Magnetic Composite	186	90 min	4-8.6	Langmuir	0.03	96	[41]
SnO_2_/CeO_2_ nano-composite	18.5	30 min	3	Freundlich and Langmuir	0.15	96.4	This work

* NR (not reported), (METAC) (methacryloyloxy)ethyl trimethyl-ammonium chloride, DNPH (2,4-dinitrophenyl hydrazine), MCM (Mobil composition of matter), LDH (layered double hydroxides), multi-walled carbon nanotubes (MWCNTs), polyaniline (PANI), poly pyrrol (PPy).
